# Sino-Canadian Collaborations in Stem Cell Research: A Scientometric Analysis

**DOI:** 10.1371/journal.pone.0057176

**Published:** 2013-02-28

**Authors:** Sarah E. Ali-Khan, Monali Ray, Dominique S. McMahon, Halla Thorsteinsdóttir

**Affiliations:** 1 Dalla Lana School of Public Health, University of Toronto, Toronto, Ontario, Canada; 2 Mississauga Academy of Medicine, University of Toronto Mississauga, Mississauga, Ontario, Canada; 3 Munk School of Global Affairs, University of Toronto, Toronto, Ontario, Canada; 4 Small Globe, Toronto, Ontario, Canada; University of Melbourne, Australia

## Abstract

**Background:**

International collaboration (IC) is essential for the advance of stem cell research, a field characterized by marked asymmetries in knowledge and capacity between nations. China is emerging as a global leader in the stem cell field. However, knowledge on the extent and characteristics of IC in stem cell science, particularly China’s collaboration with developed economies, is lacking.

**Methods and Findings:**

We provide a scientometric analysis of the China–Canada collaboration in stem cell research, placing this in the context of other leading producers in the field. We analyze stem cell research published from 2006 to 2010 from the Scopus database, using co-authored papers as a proxy for collaboration. We examine IC levels, collaboration preferences, scientific impact, the collaborating institutions in China and Canada, areas of mutual interest, and funding sources. Our analysis shows rapid global expansion of the field with 48% increase in papers from 2006 to 2010. China now ranks second globally after the United States. China has the lowest IC rate of countries examined, while Canada has one of the highest. China–Canada collaboration is rising steadily, more than doubling during 2006–2010. China–Canada collaboration enhances impact compared to papers authored solely by China-based researchers This difference remained significant even when comparing only papers published in English.

**Conclusions:**

While China is increasingly courted in IC by developed countries as a partner in stem cell research, it is clear that it has reached its status in the field largely through domestic publications. Nevertheless, IC enhances the impact of stem cell research in China, and in the field in general. This study establishes an objective baseline for comparison with future studies, setting the stage for in-depth exploration of the dynamics and genesis of IC in stem cell research.

## Introduction

Mainland China is emerging as a global leader in stem cell research [1]–[3], and as a result is becoming an increasingly attractive collaborator for international partners [4]. Since the late 1990s, development of stem cell science and technology (S&T) in China has been intense, reflecting its position as a priority area in the national government’s life sciences, innovation and biomedical development plan [1],[5]. With a focus on applied research, China is now a major global producer of stem cell research papers, has established four stem cell banks including the world’s largest cord blood stem cell bank, and is running clinical trials in the field [1],[2]. The size and forward momentum of China’s stem cell activity is underlined by the fact that in 2005, it was already estimated to have 300 researchers working on stem cell science and 30 specialized research institutes [6], while committing to spend up to $250 million on the field between 2006–2010 [3]. While China has the political will and scientific infrastructure to excel in this field, analysts have underlined the importance of international collaboration for development of this field [1]–[13] [5] [7]. Moreover, the Chinese Minister for Science and Technology, the Honourable Xu Guanhua has expressly noted the need for international support to maximize on China’s scientific investments [7]. Policies such as ‘The Outline for the Eleventh Five-Year Plan for Implementing International S&T Cooperation’, underscore China’s intention to use international partnerships to build their innovation capacity [8],[9] (see: China International Science and Technology Cooperation, http://www.cistc.gov.cn/englishversion/).

Canada has a strong stem cell research field. It has some of the leading stem cell researchers in the world, a well-coordinated national research program (see: Canadian Stem Cell Network at http://www.stemcellnetwork.ca/), and shows leadership in ethical and regulatory scholarship and oversight (see: Canadian Institutes of Health Research, Stem Cell Oversight Committee at http://www.cihr-irsc.gc.ca/e/15298.html) [10]–[12]. Nevertheless, Canada’s key strength is the quality of its scientific work. Seminal publications from Canadian researchers include the pioneering work of Till and McCulloch whose discovery of the self-renewing capacities of stem cells paved the way for establishment of the stem cell research field [13]. More recent Canadian contributions have been the discoveries of adult neural stem cells [14], retinal stem cells [15], and adult pluripotent stem cells from human skin [16]. Canada has also made advances in induced pluripotent stem cell (iPS cell) research: a group largely composed of Canadians demonstrated that mutations are strongly associated with iPS cells in early passages [17], and a team at McMaster University were the first to induce blood stem cells from fibroblasts without first passing through an embryonic-like cell state [18]. Canada’s stem cell program is smaller than China’s, with an estimated US$40M per year spent on stem cell research [19], and around 125 senior scientists working in the field [20]. However, Canada also recognizes the importance of international collaboration to remain at the leading edge of global science [21], and for the stem cell arena in particular [22]. Hence, both countries emphasize international collaboration in stem cell research and their respective strengths may perhaps be complementary, thus offering enhanced collaboration opportunities.

Scientific research endeavors are increasingly global, with numbers of internationally co-authored research papers continuing to rise [23]. Cross-border research collaboration, amongst other motivations, allows access to expertise and resources that have been developed in the partnering countries due to specific local circumstances. International scientific collaboration is generally held to boost a country’s scientific capabilities and provide opportunities for cost-sharing [24]–[26]. Moreover, studies indicate that research resulting from such collaboration garners higher than average scientific impact and increases visibility [27]–[31]. International collaboration may be particularly important in emergent and multidisciplinary domains [32], such as stem cell science that encompass basic and applied areas. Differing stem cell research regulation across nations [33], and subsequent differences in knowledge and resource access, may make collaboration particularly important in this field [34].

Previous studies have examined international collaboration in stem cell research involving the US, the UK, and countries in the Middle East [30],[35]. However, there is limited understanding of collaboration in stem cell research involving the emerging economies, particularly China. In this study we focus on China’s stem cell research collaboration with Canada. Canada and China have formally expressed their interest in working together in S&T. Their governments, for example, signed an S&T cooperation agreement in 2007, which they reiterated in 2012 by increasing funding towards collaboration (See: Government of Canada. Agreement for scientific and technological cooperation between the Government of Canada and the Government of the People’s Republic of China (2007) at http://publications.gc.ca/site/eng/364842/publication.html, and International Science and Technology Partnerships Canada at http://www.istpcanada.ca/News/CanadaChinaRDProjects.php). In discussion between Canadian and Chinese policy makers, collaboration in stem cell research was singled out as a priority area for their life science collaboration. In 2007, a few months after signing the science and technology agreement, the Canadian Stem Cell Network (SCN) and National Research Council Canada (NRC) paved the way for future partnership by sending a stem cell delegation to China and participating in the first Sino-Canadian Stem Cell Workshop (See: Stem Cell Network at http://www.stemcellnetwork.ca/celllines/december2008.php#Anchor-Chines-7276). This year, China’s Ministry of Science and Technology (MOST), the country’s primary source of public research funds, and the government of Ontario launched a bilateral Research and Innovation Fund, committing CAN$10 million to support strategic research collaborations in four key areas, of which stem cell science is one (see: Ontario Ministry of Economic Development and Innovation at http://www.mri.gov.on.ca/english/programs/ocrif/program.asp). Yet, despite such concrete action on the part of policy-makers and scientists, knowledge on the collaboration of China and Canada is lacking. There is, for example, no information available about how extensive the collaboration is already between the two countries, nor do we have insight into the key characteristics of this collaboration. This deficiency makes it difficult to know if current initiatives to promote China-Canada collaboration are successful, or to make recommendations on how to structure the initiatives so that they are most effective.

The goal of this study is to provide a detailed analysis of the extent and characteristics of scientific collaboration in stem cell research, between China and Canada. In order to do so we will put the analysis in global context and examine the levels of stem cell research publications and international collaboration in the key leading countries in stem cell research. The main objectives of the study are thus to examine:

1. Whether knowledge production in stem cell research is increasing, and who the main global producers are.
2. Who are the main collaborators globally in the stem cell field, and how the collaboration rate of China and Canada compares with other nations’.
3. The levels and key characteristics of China-Canada’s collaboration in the stem cell field.

More in depth understanding of the existing levels and characteristics of cooperation between China and Canada can better inform policy to encourage and optimize future partnership. Thus, our findings may be relevant to stem cell researchers and entrepreneurs seeking to establish and extend their international collaboration, as well as to S&T policy-makers. They may also have implications for long-term science and technology planning for China, Canada and other countries.

## Methods

For this study, we undertook a scientometric analysis of collaboration in stem cell research between China and Canada. The volume of papers concerning stem cell research published by China and Canada was derived, as well as for other leading producers in the field and from the emerging economies, for comparison. We also examined the stem cell paper outputs of the Stem Cell Network Asia Pacific (SNAP) member countries. Established in 2007, the network is now defunct, however it was active over the majority of the years examined in our study with a key goal being to foster research collaborations between member countries. We then used co-authored papers by authors from two or more countries as a proxy for international collaboration. We examined the collaboration rates of the global leaders in the stem cell field as well as of our other comparison countries. We then determined the number of Canada-China co-publications, and analyzed the collaboration preferences of the two countries by examining their top collaboration partners. Finally, we examined collaborating institutions in China and Canada, the scientific impacts of the collaboration, mutual areas of research interest as indicated by the co-publications and the funding sources credited for their collaborative work.

### Data Collection

We used the Scopus (Elsevier) database (http://www.scopus.com) to retrieve publications involving stem cell research and limited our examination to recently published papers, from 2006–2010. Scopus is an international multidisciplinary database that indexes over 17,000 peer-reviewed journals in S&T, as well as more than 500 international conference and seminar proceedings (see: http://www.info.scopus.com/scopus-in-detail/facts/, [36]. We selected Scopus for its size – it is the largest international multidisciplinary database in the world, and for its quality assurance processes – over 95% of material is peer-reviewed, and all titles are evaluated before inclusion by an external Content Selection and Advisory Board [37]. Additionally, Scopus has wider coverage of journals from developing countries and emerging economies, including China, compared to another frequently used database, the Web of Science (see: http://thomsonreuters.com/products_services/science/science_products/a-z/web_of_science/). Scopus also has better coverage from many countries where English is not the first language, and includes peer-reviewed, and evaluated non-English titles [36] (and see: http://www.info.sciverse.com/UserFiles/sciverse_scopus_content_coverage_0.pdf). Thus, analyzing data from the Scopus database should generate a relatively accurate, and the most comprehensive picture of China’s participation in the field of stem cell research.

A caveat is that some collaboration will not result in co-authored papers, while other co-authored papers may have involved only limited collaboration. Nevertheless, this methodology has been widely used [23], [27]–[30], and provides the opportunity to compare collaboration between countries, and to examine changes over time.

We used ‘stem cell’ as the search term, limiting this term to occurrence in ‘article titles, abstracts or keywords’. We solely included papers published between 2006–2010, and the search was conducted within three of the four broad subject areas offered by Scopus: life sciences (including >4,300 titles); health sciences (including >6,800 titles (100% medline coverage)); and physical sciences (including >7,200 titles). For this study, we limited our collection to three key document types that are cited by and include references to other academic publications – namely articles, conference papers and reviews – together referred to in this study as ‘papers’.

We tested various search strings before arriving at the term ‘stem cell’, which in Scopus also captures the hyphenated and plural forms (stem-cell and stem cells, respectively). To begin, we obtained a comprehensive list of vocabulary commonly used to describe stem cell research by consulting the National Library of Science’s Medical Subject Headings (MeSH). This list included: adult stem cells, embryonic stem cells, fetal stem cells, hematopoietic stem cells, mesenchymal stem cells, multipotent stem cells, myoblasts, neoplastic stem cells, neural stem cells, pluripotent stem cells, side-population cells and totipotent stem cells. We note that the term ‘stem cell’ captures all these permutations, except myoblasts and side population cells – which in some cases are not considered to be stem cells. We note that a query for ‘keywords’ in Scopus includes both author assigned keywords and index terms. The latter are assigned by Scopus indexers from standard controlled vocabularies (see: http://www.jisc-adat.com/adat/adat_db_details.pl?ns_ADAT:DB=Scopus). Author assigned keywords tend to be more specific, while index terms allow a document to be searchable in more broadly recognized and relative terms. Thus, papers mentioning more specific terms such as ‘myoblasts’ or ‘side-population cells’ in cases where they are indeed stem cells, would be likely to qualify this relationship in either the author keywords or index terms using the broadly recognizable term ‘stem cell’. Accordingly, we noted articles in our dataset captured using ‘stem cell’ such as “Side population cells isolated from hepatic carcinoma cell lines escape being killed by NK cells from NOD/SCID mice” [38], where the term ‘stem cell’ appears in both author keywords and index terms, but not in the title or abstract. Likewise, we considered a number of more and less-specific terms such as ‘progenitor cells’ before arriving at ‘stem cell’ as our search term.

Scopus search and analysis tools were used to limit the collection to papers authored by researchers affiliated to institutions in China and/or Canada. We note that throughout this work China refers to mainland China. Additionally, Scopus reports data for Taiwan and Hong Kong separately from mainland China, and we have reported their data separately likewise. By placing a limit of affiliation ‘in China’, all papers without a China-based author were excluded. Placing an additional limit of affiliation ‘in Canada’, all papers without an author affiliated in either country were excluded. These papers constituted China-Canada international collaborations. Finally, excluding affiliations in all other countries yielded papers with only Canadian and Chinese addresses. These papers constituted bilateral China-Canada collaborations, or domestic papers when all but China or Canada-authored papers were excluded. Corresponding authors were determined from the reprint address.

### Scientometric Indicators

Statistics were generated based on the following indicators using Scopus data analysis tools.

#### Volume of collaboration

The volume of collaboration was measured by the number of papers listing authors with institution affiliation addresses in countries of interest. Collaboration between countries was counted if authors with affiliation addresses from more than one country were listed. Thus, a collaboration was counted between China and Canada if at least one author from each country was listed on a paper. We calculated the number of bilateral collaborations involving exclusively Canada and China, and the number of multilateral, or international, collaborations involving Canada, China and other countries. Domestic papers were considered to be those that listed authors affiliated in only one country. The 95 China-Canada co-affiliated papers ascertained by Scopus search tools were checked manually to verify that they reported stem cell research, and that they were articles, reviews or conference papers.

#### Collaboration rate

This is an indicator of the relative importance of international collaboration for a country. It was calculated for a country by dividing the total number of papers for that country by its number of international collaboration papers.

#### Impact of collaboration

The average number of citations per paper (CPP) received for papers written in collaboration and without collaboration for each country was used as a proxy for the impact of the collaboration. Thus ‘citedness’ or CPP was calculated for a country by adding the number of citations received per paper published over the years in question, and dividing this by the total number of papers published for that country over that time. Citation rates were obtained from Scopus data using Scopus analysis tools.

#### Statistical analyses

The average numbers of CPP for domestic China papers, domestic Canada papers and for China-Canada collaborations were compared using one tailed T-Tests. Likewise, the effect of language of publication on CPP was analyzed. Some research has demonstrated a positive effect of the number of authors, affiliations, or countries participating on a paper on its ‘citedness’ [27],[39]–[41]. In light of this, it is possible that domestic papers might be less cited than bilateral papers, and bilateral papers less cited than multilateral or international collaborations. To examine whether the number of authors, affiliations or countries involved in a paper influences the number of citations it receives we performed correlation analyses. The Pearson correlation coefficient was calculated for China-Canada collaboration papers. All statistical tests were performed using Microsoft Excel 2011.

#### Salton’s measure

This is considered an indicator of the strength of mutual collaboration between two countries. It is useful because it normalizes for bias in the number of papers produced by a country because of its size. For example, the US produces far more papers than Hong Kong yet they both collaborate with China – Salton’s measure allows assessment of the relative strength of these collaborations by controlling for such differences in output [31],[42]. Salton’s measure is calculated by:
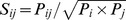



Where *P_i_* is the number of total papers of country *i*, *P_j_* is the total number of papers of country *j, and P_ij_* is the number of joint papers between both countries.

#### Mutual areas of China-Canada research interest/analysis of co-authored papers

Areas of China-Canada mutual stem cell research interest were analyzed by examining the author key words, index terms, the abstracts, and if required the body text, of the 95 co-authored papers. To gain more in-depth appreciation of the types of research carried out, we analyzed this material noting the main model system or organism under study (mouse, rat, human, other organism or theoretical/mathematical modeling study); the developmental stage of the cells under study (including embryonic, post-natal and cord blood); and the discipline or subject area under study (including angiogenesis, basic biology of stem cells – differentiation, survival, genesis etc), bio-engineering/preparation/cryopreservation/scale-up of stem cell production, cardiology, diabetes/obesity, gene therapy, heptology, imaging studies, immunology, mathematical models/theoretical studies, neuroscience, oncology, osteogenesis/bone development, public policy issues, rheumatology, other tissue engineering, and wound healing. These distinctions were made based on the key words/index terms provided, and the organ system, tissue or disease under study in each paper. We also noted whether the paper was a review or hypothesis paper, basic research, or a case study/clinical trial/applied research. This methodology was adapted from Flynn and Matthews [35].

#### Additional data

Data on funding, language and journals of publication were collected for China-Canada collaboration papers from Scopus data. This was further analyzed using Internet searches or from the papers themselves.

## Results

### Participation of Countries in Stem Cell Research

**Figure 1:**
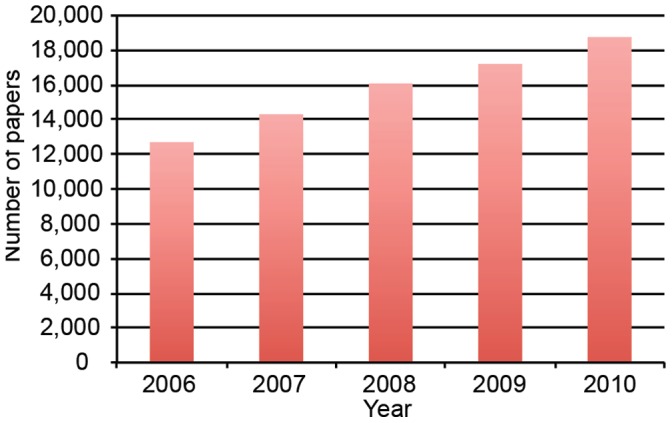
Total worldwide stem cell papers by year. Shown is the total number of papers published worldwide in stem cell research by year, 2006–2010.

In Figure 1 we present number of papers published worldwide in stem cell research between 2006–2010. Our search string generated 79,076 unique stem cell papers; 75% of these were articles, 21% were reviews and 4% were conference papers. The number of stem cell papers published globally increased from 12,700 in 2006, to 18,657 in 2010, an increase of almost 48%. Thus, our data indicate that the stem cell research field is growing rapidly. For comparison, a similar study showed a global doubling of stem cell publication numbers between 1991 and 2002 [22].

The top ten countries ranked by stem cell research paper output, and two additional emerging economies, India and Brazil, for comparison are shown in Table 1. The US dominates stem cell research over 2006–2010, with 27,378 papers published, more than three times the total published by China (8,546), the second most prolific producer over this time. Germany was the third largest producer (6,938), closely followed by Japan (6,771) and the UK (5,647). Canada was the 8^th^ largest producer (2,977). These rankings closely reiterate global rankings in overall science and engineering publications [23]. They list the top three as the US, China and Japan, with the next five countries being the same as our data, though in slightly different order. Thus, publication volume rankings in stem cell science are largely consistent with global science publication patterns. Notably, our analysis shows that in 2006, China ranked fourth, closely clustered with Germany and Japan. However, from 2007 to 2010 China’s research output rapidly pulled ahead. The other emerging economies studied here, Brazil (844–19^th^) and India (742–21^st^), did not show a particularly strong publication performance. China also out performs South Korea (2143–9^th^), another major player on the Asian stem cell scene, by almost a factor of four. Other members of SNAP – Australia (1,859), Taiwan (863), Singapore (775), and Thailand (125) – were 12^th^, 16^th^, 20^th^ and 37^th^ respectively in terms of numbers of publication volume. SNAP’s other members are China, South Korea, Japan, and India.

**Table 1:** Output of stem cell papers by top producing countries of stem cell research.

		2006	2007	2008	2009	2010	Total	% increase over 2006
1	**USA**	4524	5138	5430	5640	6646	27378	46.9
2	**China**	930	1377	1797	2055	2387	8546	156.7
3	**Germany**	1072	1239	1468	1505	1654	6938	54.3
4	**Japan**	1190	1248	1441	1394	1498	6771	25.9
5	**UK**	869	1085	1117	1231	1345	5647	54.8
6	**Italy**	638	760	920	909	1097	4324	71.9
7	**France**	579	639	712	733	812	3475	40.2
8	**Canada**	477	518	619	666	697	2977	46.1
9	**South Korea**	259	353	408	499	624	2143	140.9
10	**Spain**	292	354	391	466	563	2066	92.8
19	**Brazil**	86	123	143	226	266	844	209.3
21	**India**	92	101	137	172	240	742	160.9

Shown is a ranking of the top producing countries of stem cell research according to total stem cell papers produced between 2006 and 2010, and emerging economies Brazil and India.

Publication growth rates over the five-year study period per country are shown in Table 1. While all countries monitored show a steady increase in paper output over the years examined, China’s increase in publication rate of 156% is more than three times that of other large producers, the US (47%), Germany and the UK (54%), France (40%), Canada (46%). This is especially striking in light of China’s relatively recent entry into the stem cell research arena, and the large number of papers it now produces. Also striking were increases in publication from South Korea (141%), and from India (161%) and Brazil – which showed the largest increase over time (209%) – though in the case of the latter two countries, absolute publication output was comparatively low.

Our results reflect national policy and investment towards stem cell research over recent years. The US is the world’s largest investor in overall R&D, and so it is not surprising that it remains the world’s largest producer of stem cell research. In 2008, the US’ gross domestic expenditure on R&D (GERD) represented 42.5% of OECD countries’ total, or almost US$400B. At the same time the US accounted for 16% of the world’s scientific publishing [43]. While federal funding for human embryonic stem cell research was restricted from 2001–2008, federal funding for stem cell research in general continued to be substantial [34]. Thus, comparatively massive funding continued to push this field forward in the US.

China’s rapid rise in the stem cell arena reflects its increasing overall R&D investments, and its subsequent emergence as one of the world’s main producers of scientific knowledge. Over the decade from 2000–2010, China increased its R&D spending six-fold [8], and now represents the world’s second largest gross domestic expenditure on R&D (GERD) after the US. In parallel, between 1998 and 2008 China’s overall output of scientific papers increased by about twenty-fold [43], now ranking China third after the US and Japan in total papers published [8]. China has had a large emphasis on health research and ranks, for instance in second place globally in health biotechnology publications, just after the United States [44] Burgeoning funding and facilities, and policy to increase the number of local science trainees, retain local talent, and recruit foreign-trained China-born scientists back to China, as well as the absence of stem cell-related political and ethical controversies in China, have all contributed to its growth in the stem cell field [1],[2]. Interest seems not to be waning. This year, the national government’s funding commitment for stem cell research for the next five years was estimated at close to US$500M, a sum which does not include local government funding, industry support and other funding initiatives [1].

### International Collaboration in Stem Cell Research

**Figure 2:**
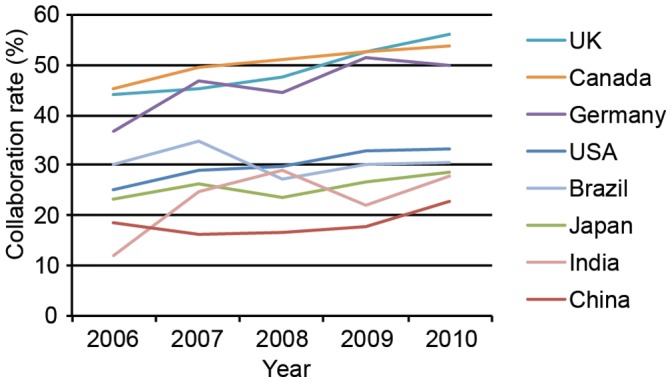
Collaboration rates of top producers of stem cell research, and Canada, Brazil and India. Shown are the collaboration rates of the top five global producers of stem cell research; the USA, China, Germany, Japan, and the UK, and for comparison - Canada, and emerging economies Brazil and India, 2006–2010.

To examine the emphasis on international collaboration in general in stem cell research, we calculated the collaboration rates in stem cell research for the leading countries that publish in the field from 2006–2010 (Table 2). We also included rates for the emerging economies Brazil and India, and for Hong Kong for comparison. Our data show a relatively large variance in collaboration rates between countries. Papers published in collaboration as a percent of total papers (average collaboration rate over time) ranged from 19% percent (China) at the lowest end of the scale to 51% (Canada, Hong Kong) at the highest. Rates of collaboration generally increased over the time period examined (Fig. 2). Of note, our data show that while China is the second most prolific producer of stem cell research in the world, it has the lowest international collaboration rate of the countries we examined.

**Table 2:** Select countries’ international collaboration rates, 2006–2010.

	Internationally co-authored papers	Domestic papers	Total papers	Collaboration rate
**USA**	8318	19059	27377	0.30
**China**	1600	6947	8547	0.19
**Germany**	3194	3745	6939	0.46
**Japan**	1754	5016	6770	0.26
**UK**	2802	2845	5647	0.50
**Italy**	1640	2684	4324	0.38
**France**	1640	1836	3476	0.47
**Canada**	1514	1463	2977	0.51
**South Korea**	595	1548	2143	0.28
**Spain**	914	1152	2066	0.44
**Brazil**	257	587	844	0.30
**India**	181	561	742	0.24
**Hong Kong**	204	193	397	0.51

Shown is the collaboration rate (i.e. papers written in international collaboration as a percentage of the total stem cell research papers produced) of top producing countries of stem cell research, as well as for comparison - Hong Kong, and emerging economies Brazil and India.

Commentators have underscored the importance of international collaboration in advancing stem cell science. For example, international collaboration has been a key strategy in diversifying and progressing research by the California Institute of Regenerative Medicine [45]. Canadian analysts have pointed to the role of international partnerships for Canada’s advance on the global stem cell scene [22]. International collaboration with China-born western-trained scientists has been instrumental in some of China’s key stem cell accomplishments [1]. Smaller or less scientifically productive nations have been noted to seek higher rates of international collaboration [46],[47]. Nevertheless, our data show that in the stem cell field, relatively large countries with strong stem cell research programs (Germany, UK, France, Canada) published about 50% of their research in collaboration.

The US has been shown to be a preferred partner in overall scientific collaboration with most nations [23] [23] [47]. We also show it is the top collaboration partner for both China and Canada in stem cell research. With ample infrastructure, funding and human resources of its own, the US has more modest collaboration rates (30%). Nevertheless, others have shown it also benefits in terms of citation rate by engaging in international stem cell research collaborations [30]. Research indicates that China’s relative international collaboration in science is decreasing as its scientific capacity and output increase [42],[48]. Our data show that in the stem cell field, its relative collaboration rate is low and stable.

### China and Canada’s Key International Collaborators in Stem Cell Research

**Figure 3:**
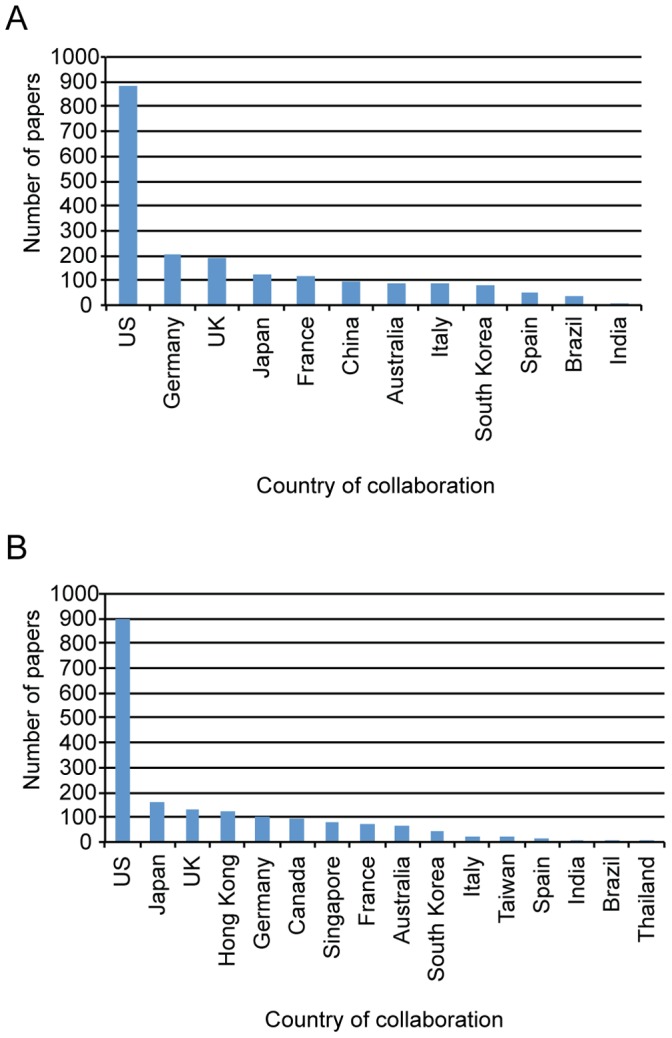
Canada’s and China’s total collaboration papers with key producers of stem cell research. A: Canada’s collaboration papers, 2006–2010. Shown are Canada’s total numbers of collaboration papers with the other top nine global producers of stem cell research, and for comparison - emerging economies Brazil and India, and Australia. **B:** China’s collaboration papers, 2006–2010. Shown are China’s total number of collaboration papers with the other top nine global producers of stem cell research, and for comparison - emerging economies Brazil and India, Stem Cell Network Asia Pacific (SNAP) members, and Hong Kong.

**Figure 4:**
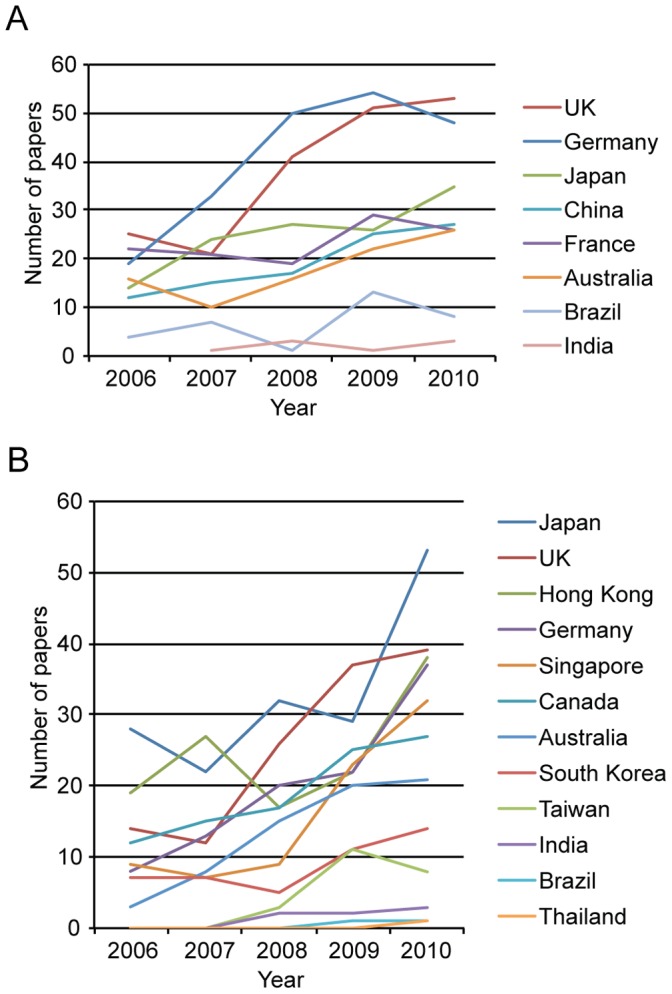
Canada’s and China’s collaboration with their top five collaboration partners, and select others. A: Top Canada’s collaboration (collaboration with US not shown). Shown is the number of stem cell papers co-authored by Canada per year, together with its top five collaboration partners after the US (its top collaborator – data not shown); Germany, the UK, Japan, France and China, as well as for comparison - the number of co-authored papers with Australia, and emerging economies Brazil and India. **B:** Top China’s collaboration (collaboration with US not shown). Shown is the number of stem cell papers co-authored by China per year, in collaboration with its top five collaboration partners after the US (its top collaborator – data not shown); Japan, the UK, Hong Kong, Germany, and Canada, as well as for comparison - the number of co-authored papers with Stem Cell Network Asia Pacific (SNAP) members, Hong Kong, and emerging economies Brazil and India.

We examined the top collaboration partners of Canada and China by examining more closely their co-authored papers produced between 2006–2010 (see Figures 3A, 3B and 4A, 4B). The results showed that the US was by far the top partner for both countries, showing 885 co-affiliated papers with Canada, and 901 with China over this period. For Canada, the top collaborators in terms of papers are Germany (204), the UK (191), Japan (126), France (117), China (96) and Australia (90). For China, we also examined collaboration with SNAP member countries, and with Hong Kong because of their historical, cultural and political ties. China’s top collaborators in terms of numbers of papers after the US were Japan (164), the UK (128), Hong Kong (123), Germany (100), Canada (96) and Singapore (80). Thus, Canada ranked as 6^th^ key partner for China, and vise versa for China’s collaboration with Canada, demonstrating a relative symmetry in the emphasis the two countries place on collaboration with each other in terms of numbers of papers published. Our rankings for stem cell research papers very closely reflect China’s international collaboration partners for overall scientific publication [42]. Examining Canada’s numbers over time (Figure 4A) shows overall increased numbers of collaborative papers with most countries, notably Germany, Japan, China and the UK. Increases in the number of collaborations with these countries were 153%, 150%, 125% and 112% respectively. China (Fig. 4B) also showed increased numbers of co-affiliated papers over time with almost all countries examined, in particular with Australia (600%), Germany (363%), the US (309%), Singapore (225%), the UK (179%), and Canada (125%).

We next examined the collaboration preferences of countries, while controlling for the bias arising from the varying numbers of papers published by different countries using Salton’s measure. Salton’s is a representation of the mutual strength of collaboration between the two countries – the larger the value of Salton’s measure, the stronger is the collaboration preference. To examine the strength of collaboration in stem cell science for China and Canada, and their collaboration partners we calculated Salton’s measures over 2006–2010 (Table 3, Table 4). For Canada, these clearly indicate the strongest mutual preference for collaboration of the countries we examined is with the US (0.097), followed by the UK (0.045), Germany (0.044), Australia (0.037) and France (0.037). China ranked 10^th^ in Canada’s preference for partners in stem cell research collaboration of the countries we examined (0.019). On China’s side, Salton’s measure for Hong Kong was highest (0.069), followed by the US (0.056), Japan (0.030), Singapore (0.029), and then Canada (0.019) in 5^th^ position of our selected countries. These results reflect an effect on collaboration preference of geographical proximity, and also – particularly in the case of Hong Kong and China, and for Canada with the UK, Australia and France – historical, cultural and linguistic ties. Clearly for both China and Canada, there is much to be gained from taking advantage of US strengths in stem cell science. Thus far, China’s collaboration with India and Brazil has been limited, indicated both by numbers of co-affiliated paper numbers and Salton’s measure respectively; with India (7 papers 2006–2010, Salton’s measure 0.002) and Brazil (2 papers, 0.0004). This situation may shift in the future as these countries recently committed to collaboration in R&D, innovation and technology transfer toward improving public health (BRICS Health Ministers’ Meeting- Beijing Declaration, 2011, see: http://keionline.org/node/1183). Nevertheless, while China’s stem cell program is still relatively young, there may be greater advantage in accessing scientific knowledge and building international reputation through collaboration with established players, rather than partnering with other newcomers to the field.

**Table 3:** Canada’s strength of collaboration with other top producers of stem cell research, 2006–2010.

	2006	2007	2008	2009	2010	Average
**US**	0.089	0.096	0.093	0.105	0.104	0.097
**UK**	0.039	0.028	0.049	0.056	0.055	0.045
**Germany**	0.027	0.041	0.052	0.054	0.045	0.044
**Australia**	0.044	0.024	0.033	0.041	0.044	0.037
**France**	0.042	0.037	0.029	0.042	0.035	0.037
**South Korea**	0.026	0.026	0.036	0.040	0.024	0.030
**Japan**	0.019	0.030	0.029	0.027	0.034	0.028
**Italy**	0.020	0.018	0.025	0.028	0.025	0.023
**Brazil**	0.020	0.028	0.003	0.034	0.019	0.021
**Spain**	0.011	0.023	0.012	0.023	0.029	0.020
**China**	0.018	0.018	0.016	0.021	0.021	0.019
**India**	0.000	0.004	0.010	0.003	0.007	0.005

Shown is the strength of Canada’s collaboration (indicated by Salton’s measure – the larger the number, the stronger the mutual strength of the collaboration between the two countries) with the other top 10 global producers of stem cell research, as well as for comparison – Australia, and emerging economies India and Brazil.

**Table 4:** China’s strength of collaboration with other top producers of stem cell research, 2006–2010.

	2006	2007	2008	2009	2010	Average
**Hong Kong**	0.081	0.083	0.049	0.056	0.074	0.069
**US**	0.038	0.043	0.056	0.062	0.081	0.056
**Japan**	0.027	0.017	0.020	0.017	0.070	0.030
**Singapore**	0.030	0.017	0.018	0.037	0.043	0.029
**Canada**	0.018	0.018	0.016	0.021	0.021	0.019
**UK**	0.016	0.010	0.018	0.023	0.022	0.018
**Australia**	0.006	0.012	0.018	0.021	0.019	0.015
**France**	0.016	0.009	0.016	0.015	0.011	0.014
**Germany**	0.008	0.010	0.012	0.013	0.019	0.012
**South Korea**	0.014	0.010	0.006	0.011	0.011	0.010
**Taiwan**	0.000	0.000	0.005	0.016	0.011	0.007
**Italy**	0.004	0.003	0.002	0.007	0.003	0.004
**Spain**	0.004	0.003	0.002	0.006	0.001	0.003
**India**	0.000	0.000	0.004	0.003	0.004	0.002
**Thailand**	0.000	0.000	0.000	0.000	0.003	0.001
**Brazil**	0.000	0.000	0.000	0.001	0.001	0.0004

Shown is the strength of China’s collaboration (indicated by Salton’s measure – the larger the number, the stronger the mutual strength of the collaboration between the two countries) with the other top 10 global producers of stem cell research, as well as for comparison – the Stem Cell Network Asia Pacific (SNAP) members, Hong Kong, and emerging economies India and Brazil.

### China-Canada Collaboration

**Figure 5:**
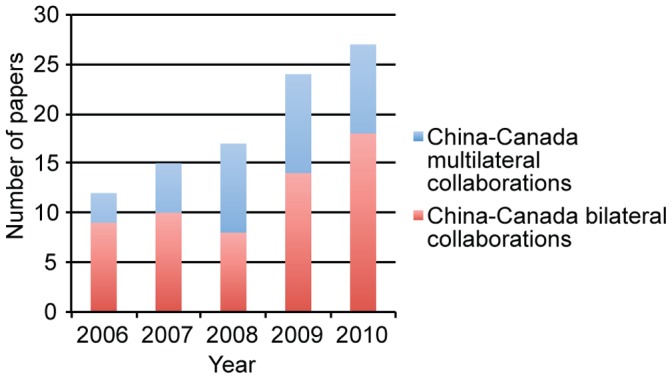
Total China-Canada collaboration papers written per year. Shown is the total number of stem cell papers co-authored by China and Canada, 2006–2010**.** The number of bilateral collaboration papers between the two countries is shown in red, while the number of multilateral collaboration papers, involving other countries in addition to China and Canada, is shown in blue.

We focus on yearly trends in China-Canada co-publications in stem cell research in Figure 5. We see that collaboration between the two countries is on a steady rise. Canada-China collaborations in stem cell research have increased from year to year, more than doubling between 2006–2010 for a total of 95 papers; 84% articles, 14% reviews, and 2% conference papers. Of these, 62% were bilateral collaborations involving only China and Canada. Bilateral collaborations also increased over time, for a total of 59 papers; 86% articles, 12% reviews, and 0.2% conference papers.

China and Canada have had over 130 years of interaction, including commerce, immigration and missionary ventures [49]. One example of amicable relations between the countries is the story of Canadian physician Norman Bethune, a figure of historical importance in China for contributions made to the field of health care delivery. In 2009, the Canadian Institutes of Health Research (CIHR) and the China Scholarship Council (CSC) initiated a Canada-China Norman Bethune Health Research Scholarship Program, offering Chinese doctorate students the opportunity for health-related training in Canadian institutions (see: http://www.cihr-irsc.gc.ca/e/39515.html). Such programs aim to facilitate engagement between scientists from the two countries, allowing them to build relationships and embark on collaborative ventures.

Politically, Canada and China have witnessed fluctuations in the tone of their bilateral relationship [49]. However, both countries seem to recognize the mutual political and economic advantages of further partnership, and the Chinese and Canadian scientific communities seem to be increasing their joint output in stem cell publications.

Of the 95 international China-Canada collaborations, 50% listed an author-based at a Chinese institution as the corresponding author, 31% at a Canadian institution, and 14% were US-based. Of the 59 bilateral China-Canada collaborations, 66% listed China-based corresponding authors, and 34% were Canada-based. These results suggest that China-based researchers are driving these bilateral and international collaborations.

### Impact of Collaboration

We examined the impact collaboration is having on the scientific community by calculating the average number of CPP for stem cell research papers published between 2006–2010. The CPP for domestic Chinese papers (listing China-affiliated researchers only) was 4.22. In contrast, the CPP for domestic Canadian papers was 16.99, a highly significant difference (P<0.001). For papers listing both Chinese and Canadian authors (international or multilateral China-Canada collaborations) the CPP was 15.11. Thus, collaboration with Canada dramatically increases the average citation rate for China-based researchers (P>0.001). The decrease in average citation rates for Canada-based authors in collaboration with Chinese authors was small and not significant. Papers authored in bilateral collaboration between China and Canada showed a lower CPP (11.7) compared to multilateral China-Canada collaborations (20.2), however this difference was not statistically significant. Some research has shown that a publication’s ‘citedness’ almost solely depends on the number of affiliations or authors involved [27],[39]–[41]. To discern the effect of internationalization and cooperation on impact, as opposed to the number of authors or affiliations, we calculated the Pearson correlation coefficients for the relationship between the number of citations received by a paper and the number of authors, affiliations and number of countries listed on the papers. For both bilateral and multilateral China-Canada collaboration, there was very low correlation between number of authors (R = 0.07), number of affiliations (R = −0.06), or number of countries (R = 0.08) and the number of citations a paper received. Thus, our analysis indicates that the increase in impact observed in China-Canada collaboration is due to internationalization or cooperation, rather than the number or authors, affiliations or countries involved on a paper.

All 95 China-Canada collaboration papers were written in the English language. In contrast, of domestic papers out of China (6948), 53% were in English, 48% were in Chinese, and 0.13% in Japanese, with the remaining few published in French. A total of 99.6% of domestic Canadian papers (1467) were in English. English is the primary language of global science, and publication in English greatly increases the number of researchers able to access, and thus cite, a paper. Thus, we examined the effect of publication language on ‘citedness’ for domestic Chinese papers. The CPP for papers published in Chinese was 0.47. In contrast, for those published in English the CPP was 7.64 (P>0.001), suggesting a significant effect of publication language on impact. We also compared the CPP of domestic Chinese papers published in English with China-Canada collaboration papers. The highly significant effect on impact of collaboration with Canada-based researchers for China-based researchers remained even when the effect of language was taken into account (P = >0.001). Alongside accessing complementary or novel expertise, an ancillary benefit Chinese researchers may gain in working with Canadian counterparts is the production of high quality manuscripts in English; publication in English is an important route by which non-English speaking nations can bring their research into the mainstream.

### Publication Journals

To further examine the characteristics of the 95 China-Canada collaboration papers, we looked at the journals in which researchers are choosing to publish their research (Table S1). The 95 papers were published in 76 different journals. The greatest number was published in PLoS One, reflecting a preference for open access and a journal focusing on a wide spectrum of scientific disciplines. Publication in such a journal may optimize the dissemination of research findings to other scientists in the field, making it freely available to stakeholders in both high and low income settings. This too may be a strategy by which Chinese scientists are facilitating greater international recognition for their work.

### Funding on China-Canada Collaboration Papers

We investigated the funding sources credited in the 95 China-Canada collaboration papers. The National Natural Science Foundation of China (NSFC) was the top funder credited, appearing on more than 25% of the papers, followed by the Canadian Institutes of Health Research (CIHR), which was listed on almost 17%. Just over 80% percent of papers for which funding information was available were supported at least in part by the NSFC, China’s MOST through their National ‘863’ applied research or their ‘973’ basic research funding programs, National State Key labs program – laboratories sanctioned by the Chinese government to pursue areas of particular national interest – or local Chinese city or provincial S&T bureaus. Likewise, more than forty percent of papers acknowledged support from federal or provincial Canadian S&T programs including the CIHR, National Sciences and Engineering Council (NSERC), Canadian Foundation for Innovation (CFI), the Canada Research Chairs program, the Fonds de la Recherche en Santé du Québec (FRSQ), and the Canadian National Research Council (CNRC). Several studies were also partially funded by more targeted research funding bodies, such as the Canadian Stem Cell Network and by national or provincial disease specific groups such as the Heart and Stroke Foundation of Canada, the American Cancer Society, and the Leukemia & Lymphoma Society of Canada. Many of these bodies specifically mention nurturing international collaboration as being an important value or goal (for examples see: Internationalization of CIHR funding policy and program tools at http://www.cihr-irsc.gc.ca/e/40953.html; National Sciences and Engineering Research Council of Canada, International Opportunities at http://www.nserc-crsng.gc.ca/International-Internationale/Index_eng.asp; MOST, International Cooperation at http://www.most.gov.cn/eng/cooperation/200610/t20061008_36195.htm; Shanghai Municipal Science and Technology Commission at http://www.shanghai.gov.cn/shanghai/node17256/node17679/node17681/userobject22ai12991.html; NSFC, International Cooperation and Exchange at http://www.nsfc.gov.cn/e_nsfc/desktop/zn/0110.htm). Among these agencies, for example, the NSFC and CIHR established the China-Canada Joint Health Research Initiative grants program in 2005 to foster research collaboration between China and Canada. The CIHR and NFSC contribute US$1,138,000 and US$800,000 respectively per year for this program (see: Research Net, Funding Opportunity Details at http://www.researchnet-recherchenet.ca/rnr16/vwOpprtntyDtls.do?prog=1520&view=&type=AND&resultCount=25). Joint dedicated funding is an important incentive for encouraging international collaboration between research communities. This initiative is planning to support projects in multiple areas. Stem cell science is not highlighted, although such projects could fit under some of the six focus areas. Thus, accessible funding for China-Canada stem cell research collaboration exists. Such collaboration may be further enhanced by dedicated stem cell-targeted initiatives.

No paper credited the International Science and Technology Partnerships Canada (ISTP) initiative, which has been given the task of delivering the Canadian government’s commitment to promote science and technology collaboration with China. Accordingly, projects in stem cell research are not yet among those seventeen projects funded through ISTP (see: ISTP Canada, China-Canada R&D projects at http://www.istpcanada.ca/international_programs/China/current_projects/index.php). Also none of the 95 China-Canada collaboration papers cited any international collaboration-specific funding by name.

### Collaborating Institutions

To begin to understand more about the groups and institutions in Canada and China who chose to collaborate, we examined the affiliations of authors on collaborative stem cell science papers (data not shown). According to Scopus results, the top five Canadian institutions from which this research is produced are the University of British Columbia (Vancouver), the University of Calgary (Calgary), the University of Toronto (Toronto), the Universities of Manitoba and the University of Alberta (Edmonton). The top 4 Chinese institutions were Tsinghua University (Beijing), Shanghai Jiaotong University (Shanghai), Harbin Medical University (Harbin), and Third Military Medical University (Chongqing). We also examined the top authors producing co-affiliated papers (data not shown). These data suggest that China’s international collaborations may often be established through colleagues based in Canada or the US, who may have been educated in, or have national ties to, China. For example, B. Zhou and M-C Poon were the two top authors in terms of numbers of papers published. Both authors are affiliated at the National Research Center for Stem Cell Engineering and Technology in Tianjin, while Poon is also affiliated at the University of Calgary. Examination of their publications reveals they have published together multiple times between 2006–2010. Both Zhou and Poon have published with multiple colleagues at the National Research Center for Stem Cell Engineering and Technology, as well as others affiliated at other Chinese institutions. On several papers Zhou is also affiliated at Harvard Medical School, and has published with colleagues there on several papers. These data begin to suggest how collaborative relationships may be sparked and propagated through groups and institutions, and could be explored and verified through further in depth study.

### Areas of Mutual Research Interest

Finally, to ascertain areas of China-Canada mutual stem cell research interest and to gain deeper insight on the type of projects being under-taken by collaborating researchers, we analyzed the author key words, index terms, the abstracts, and if required the body text, of the 95 co-affiliated papers. We also examined the country of affiliation of the corresponding author on these papers to begin to gain some perspective on the country driving the research in question (Table S2).

There were 70 basic research papers. Canada-China basic research collaborations covered a range of disciplines with the greatest number of papers focused in oncology, studies of basic stem cell biology, neuroscience, cardiology, and mechanisms of bone development/osteogenesis. The majority of the basic research papers utilized adult cell types (40), followed by mouse embryonic stem cells. Only three studies focused on manipulations with human embryonic stem cells (hESCs), with the corresponding author for these papers based in Canada. The relatively low number of studies on hESCs was perhaps unexpected given that regulatory guidelines on these cell-types are less restrictive in China, and it has been suggested that international collaborators may target hESC research in seeking collaborations in Asia [30]. Conversely, it may be beneficial for Chinese researchers to collaborate on such research with countries with tighter regulation, such as Canada, in order to build public trust – particularly international trust – in their work.

There were nine papers reporting clinical trial or case reports featuring allogenic or autologous stem cell transfers. The corresponding author for five of these was China-based, while three were US-based. Six of these studies were carried out in China, one reported worldwide multi-centre data, and there was no information on where the trial took place for two. Analysts have noted that the availability of enormous patient populations in China allowing for faster and less costly clinical trials may be another attraction for international stem cell research collaborators [3],[50]. However, robust regulatory mechanisms that can deal with the rising international interest in collaboration are needed [3].

## Discussion

The aim of this study was to examine the levels and characteristics of China-Canada stem cell research collaboration, placing these in the context of China and Canada’s collaboration with other global leaders in the field. International collaboration has been fundamental to the advance of stem cell research, a field characterized by asymmetries in knowledge and capacity between nations, as well as marked variation in regulatory policy. Additionally, international discourse and networks are key features of this field. Trans-border knowledge transfer resulting from international collaboration is becoming increasingly important for the scientific and economic expansion of nations, accelerates knowledge creation, and can enhance political understanding between countries. Thus, encouraging these relationships has become a key component of many countries’ biomedical and development policy [23], [25],[26].

Our results show a global increase in knowledge production in the stem cell field, characterized by highly collaborative research endeavors. Based on our data, China is now the second largest global producer of stem cell research after the US, and is showing rapid and sustained growth in publication rate where other countries appear to have reached a plateau. China thus seems well positioned to influence global developments in the field, and given their focus on application [3], also in therapy and technology. However, Chinese stem cell research endeavors have encountered some skepticism from the global scientific community [2]. Fortifying public trust, particularly internationally, has been highlighted as a key area in need of attention [3]. Collaboration with international partners, for example Canada, may assist in bringing Chinese research into the mainstream by building its credibility. Canada is the 8^th^ largest global producer of stem cell research and, as discussed above, has made some key cutting edge contributions to the field.

Despite China’s intentions to expand international collaboration [7],[8], our analysis shows it has the lowest stem research collaboration rate of the countries examined. Moreover, this value is lower than China’s overall international science co-authorship rate (25%) [23]. By comparison, other strong research nations – namely Canada, France, Germany and the UK – co-authored about half of their research internationally. Thus, while China is being increasingly courted to engage in international S&T collaboration, most of China’s development in stem cell research is occurring within China. Nevertheless, China’s collaboration with Canada in stem cell research more than doubled from 2006 to 2010, although it remains relatively modest.

The phenomena underlying China’s low international collaboration may hinge on paucity of international trust in China’s research endeavours, poor inter-country communication due to lack of language skills, or simply limited international knowledge of Chinese labs, due to China’s short history in the stem cell arena. Nevertheless, multiple opportunities to increase Chinese collaboration exist. For example, within Asia China’s collaboration with former SNAP members seems low, particularly with South Korea which shares similar stem cell research aspirations, Singapore with its Biopolis development, and India, which has a rapidly developing health biotechnology scene [51],[52]. Our data also point to low research engagement of Canada with India and with Brazil, countries that are also building capacity in the field [51],[53].

Our analysis indicates China-Canada collaboration greatly enhances scientific impact compared to papers authored solely by China-based authors. The difference remained highly significant even when comparing only papers published in English and thus cannot be attributed to publication language. Our data also shows that the increase in impact observed for China-Canada collaborations is not due to the number of authors, affiliations, or number of countries involved in the collaboration. Instead, cooperation and internationalization of the research are key factors. It is therefore of mutual interest for countries such as Canada and China to collaborate with one other. Canada gains access to extensive knowledge production in China, while China’s researchers benefit from augmented scientific impacts.

We show that China-Canada collaboration is largely shaped by the bilateral component, as more than half of the interactions are of this type. In terms of scientific impact, bilateral collaborations were no less beneficial than multilateral collaborations. The majority of China-Canada co-authored papers list a China-based corresponding author, suggesting that China-Canada collaborations are mainly driven or initiated from the Chinese side. Part of the reason may be the high number of China-born students who travel to North America, including Canada, for training. Many end up staying, and may initiate research relationships with counterparts in their home countries. National Science Foundation (NSF) data indicates that more foreign-born faculty participate in international research collaboration (34%) than those born in the US (28%) [23]. Diaspora is becoming increasingly important in the establishment of business and research links [54],[55]. Canada already has high numbers of China-born immigrants and students, and the flow of Chinese students to Canada is likely to increase following recent initiatives to align Chinese secondary education with Canadian curricula (see: http://news.xinhuanet.com/english/culture/2012-02/11/c_131404525.htm, and http://www.asiapacific.ca/media/press-releases/3468). Thus, Canada and China are well-placed to exploit these channels in building their collaboration. The effects of this educational policy on R&D and business collaboration between China and Canada should be followed and studied in depth. However, it appears more researcher/student flow occurs from China to Canada than vise versa, and this may underlie the asymmetry in corresponding authorship in China-Canada collaborations. Increasing this flow in the Canada to China direction may have a marked effect on collaboration. Programs to foster this flow should be expanded and their effects on collaboration studied. Such programs could include increased Canadian student exchanges to China, coupled with opportunities to study Chinese language at universities in Canada.

Recently, a number of programs to fund China-Canada S&T cooperation have been initiated or reiterated. Establishing bilateral agreements between Chinese and Canadian funding agencies to provide dedicated support for partnerships in stem cell research can enhance engagement between the research communities on both sides. However, thus far there is little explicit targeting of stem cell research, and this should be remedied in order to optimize collaboration and energize mutual development of the field in China and Canada. Networking events such as joint symposia, and exploration initiatives – in both directions – such as the aforementioned 2008 Canadian stem cell delegation’s trip to China should be increased (See: Stem Cell Network at http://www.stemcellnetwork.ca/celllines/december2008.php#Anchor-Chines-7276). Additionally, it is important that all institutional actors – funding agencies, technology transfer offices, intellectual property experts, etc – that are involved in supporting stem cell research engage with counterparts in collaborators’ countries. Encouraging such institutional engagement and alignment can play an important role in not only bringing about cross-border interoperability in terms of policies, but also to build trust between countries, as well as to optimize the efficiency and effectiveness of collaborative activities. This may further facilitate awareness of Chinese stem cell research by the international scientific community, and aid in achieving greater international recognition.

Several limitations should be noted in interpreting our study. Firstly, although the rankings of countries’ stem cell research output in our data closely reiterate overall rankings in global science publications [23], countries’ paper numbers are greater than some previous stem cell research estimates [1] [2],[56]. This may partially be a function of our use of the Scopus database, which includes 38% more journals than Thompson’s ISI, and more non-English language publications [36]. Although these previous studies have not published extensive methodology for their results, they may also have examined a different combination of publication types or subject areas of focus. Additionally, China has a larger number of domestic journals (some publishing in Chinese, some in English) than many other countries, thus publication in such journals may also contribute to our higher estimations. A second and key limitation of this study is the fact we can only speculate on the phenomena that underlie our scientometric results. Detailed case study research should be employed to further examine the factors and conditions that shape these collaborations.

In summary, this study provides an analysis of the extent and characteristics of stem cell research collaboration between China and Canada 2006–2010, relying on scientometric data. It establishes an objective baseline against which future studies on China, Canada and other countries can be compared. It also sets the stage for in-depth study of the detailed dynamics and genesis of collaboration between China and Canada, with the goal of gathering specific information about how to encourage these relationships. As research becomes increasing trans-national and funding agencies target collaboration, identifying strategies for successful collaboration will be essential for advancing stem cell science, realizing its promise for improving global health [57], and making efficient use of the resources available.

## Supporting Information

Table S1 Journals of publication of China-Canada collaboration papers. Shown are the journals in which the 95 China-Canada collaboration papers written from 2006–2010 were published, and the impact factors of these journals.
(DOCX)

Table S2 Overview of China-Canada stem cell research collaboration 2006–2010 based on co-authored papers. Shown are the areas of mutual interest of China and Canada in stem cell research, as indicated by an analysis of the author keywords, index terms, the abstracts and if required the body text of the 95 co-affiliated papers written from 2006–2010. We note the area of study of each co-authored paper, the model system or cell lines, and the developmental stage of the cells utilized in the research. Finally, to begin to gain perspective on which country may be driving each collaboration, the affiliation country of the corresponding author involved in the collaboration papers are listed.
(DOCX)
