# Supplementary material for: Sino-Canadian Collaborations in Stem Cell Research: A Scientometric Analysis
Source: PLoS One. 2013 Feb 28;8(2):e57176. doi: 10.1371/journal.pone.0057176 (PMC3585304; doi:10.1371/journal.pone.0057176)
Supplement: Table S1 — Journals of publication of China-Canada collaboration papers. Shown are the journals in which the 95 China-Canada collaboration papers written from 2006–2010 were published, and the impact factors of these journals. (DOCX) [file pone.0057176.s001.docx]

**Table S1: Journals of publication of China-Canada collaboration papers**

| **Journal name** | **Impact factor (2010)** | **# of papers** |
| --- | --- | --- |
| PLoS One | 4.411 | 4 |
| Journal of Biological Chemistry | 6.914 | 3 |
| Medical Hypotheses | 1.389 | 3 |
| Stem Cells | 7.871 | 3 |
| Stem Cells and Development | 4.791 | 3 |
| American Journal of Physiology Heart and Circulatory Physiology | 3.88 | 2 |
| Cytotherapy | 2.925 | 2 |
| Cell Biology International | 1.747 | 2 |
| Journal of Cellular Biochemistry | 3.122 | 2 |
| Journal of Thrombosis and Haemostasis | 5.439 | 2 |
| Journal of Translational Medicine | 3.51 | 2 |
| Proceedings of the National Academy of Sciences of the United States of America | 9.977 | 2 |
| All other journals each with one paper |  | 65 |
