# Supplementary material for: Sino-Canadian Collaborations in Stem Cell Research: A Scientometric Analysis
Source: PLoS One. 2013 Feb 28;8(2):e57176. doi: 10.1371/journal.pone.0057176 (PMC3585304; doi:10.1371/journal.pone.0057176)
Supplement: Table S2 — Overview of China-Canada stem cell research collaboration 2006–2010 based on co-authored papers. Shown are the areas of mutual interest of China and Canada in stem cell research, as indicated by an analysis of the author keywords, index terms, the abstracts and if required the body text of the 95 co-affiliated papers written from 2006–2010. We note the area of study of each co-authored paper, the model system or cell lines, and the developmental stage of the cells utilized in the research. Finally, to begin to gain perspective on which country may be driving each collaboration, the affiliation country of the corresponding author involved in the collaboration papers are listed. (DOCX) [file pone.0057176.s002.docx]

**Table S2: Overview of China-Canada stem cell research collaboration 2006 – 2010 based on co-authored papers**

| **Paper type** | **Discipline/area of study** |  | **Model system/cell lines** | **Developmental stage of cells under study** | **Country of corresponding author** |
| --- | --- | --- | --- | --- | --- |
| **Basic research articles** | Angiogenesis | 6 | 1 rat  1 human  4 mouse | 5 adult  1 post-natal | 3 China  1 Canada  2 US |
|  | Basic stem cell biology/survival/differentiation/genesis | 11 | 2 rat  1 human  6 mouse  2 other | 7 adult  3 embryonic (of which 1 human embryonic stem cell (hESC)  1 post-natal/cord blood | 5 China  5 Canada  1 Germany |
|  | Bio-engineering/preparation/cryopreservation/scale-up of stem cell production | 2 | 2 human | 2 hESC | 2 Canada |
|  | Cardiology | 8 | 5 rat  3 mouse | 3 adult  3 embryonic  2 post-natal | 2 China  5 Canada  1 US |
|  | Diabetes/obesity | 1 | 1 mouse | 1 adult | 1 China |
|  | Gene therapy | 1 | 1 mouse | 1 adult | 1 China |
|  | Hepatology (differentiation path to hepatocytes) | 1 | 1 rat | 1 adult | 1 Canada |
|  | Imaging studies | 1 | 1 rat | 1 adult | 1 Canada |
|  | Mathematical models/theoretical studies | 3 | N/A | N/A | 1 China  1 Canada |
|  | Neuroscience | 10 | 5 rat  5 mouse | 4 adult  5 embryonic  1 post-natal | 6 China  3 Canada  1 USA |
|  | Oncology | 12 | 5 human  6 mouse  1 n/a | 7 adult  2 embryonic  2 post-natal/cord blood 1 n/a (clinical guidelines) | 2 China  4 Canada  4 US  1 Italy  1 France |
|  | Osteogenesis/bone development | 8 | 3 rat  3 mouse  2 other | 5 adult  1 embryonic  2 post-natal/cord blood | 8 China |
|  | Other tissue engineering | 2 | 2 mouse | 2 adult | 1 Germany |
|  | Public policy issues | 1 | n/a | induced pluripotent stem cells | 1 Canada |
|  | Wound healing | 3 | 1 human  2 mouse | 3 adult | 2 China  1 Canada |
|  | **Total** | **70** |  |  |  |
| **Reviews** | Angiogenesis | 3 |  | N/A | 3 China  1 US co-affiliation |
|  | Cardiology | 2 |  | N/A | 2 Canada |
|  | Diabetes/obesity | 1 |  | N/A | 1 China |
|  | Gastro-enterology | 1 |  | N/A | 1 Canada |
|  | Hepatology/liver disease | 1 |  | N/A | 1 China |
|  | Oncology | 2 |  | N/A | 2 Canada |
|  | Orthpaedics/arthritis | 1 |  | N/A | 1 China |
|  | Wound healing | 2 |  | N/A | 2 China |
|  | **Total** | **13** |  |  |  |
| **Hypotheses** | **Total** | **3** |  | N/A | 2 China; 1 USA |
| **Clinical trials/case studies** | Angiogenesis | 1 | 1 autologous transfer | 1 adult | 1 China |
|  | Cardiology | 1 | 1 autologous transfer | 1 adult | No info available |
|  | Neuroscience | 4 | 2 autologous 2 allogenic transfers | 3 adult; 1 cord blood | 3 China  1 US |
|  | Oncology | 2 | 1 autologous 1 allogenic transfer | 1 adult | 1 China  1 US |
|  | Rheumatology | 1 | 1 autologous transfer | 1 adult | 1 US |
|  | **Total** | **9** |  |  |  |
|  | **Total collaboration papers** | **95** |  |  |  |
